# Incidence of Sleep Bruxism in Different Phenotypes of Obstructive Sleep Apnea

**DOI:** 10.3390/jcm11144091

**Published:** 2022-07-14

**Authors:** Joanna Smardz, Mieszko Wieckiewicz, Anna Wojakowska, Monika Michalek-Zrabkowska, Rafal Poreba, Pawel Gac, Grzegorz Mazur, Helena Martynowicz

**Affiliations:** 1 Department of Experimental Dentistry, Wroclaw Medical University, 50-425 Wroclaw, Poland; joannasmardz1@gmail.com; 2 Department and Clinic of Internal Medicine, Occupational Diseases, Hypertension and Clinical Oncology, Wroclaw Medical University, 50-556 Wroclaw, Poland; ania.wojakowska@wp.pl (A.W.); monika.michalek1@gmail.com (M.M.-Z.); sogood@poczta.onet.pl (R.P.); grzegorzmaz@yahoo.com (G.M.); helenamar@poczta.onet.pl (H.M.); 3 Department of Population Health, Division of Environmental Health and Occupational Medicine, Wroclaw Medical University, 50-345 Wroclaw, Poland; pawelgac@interia.pl

**Keywords:** sleep bruxism, obstructive sleep apnea, sleep-related breathing disorder, sleep body position, rapid eye movement sleep

## Abstract

(1) Background: Sleep bruxism (SB) is a common sleep behavior. Obstructive sleep apnea (OSA) is a prevalent sleep-related breathing disorder with potential long-term major neurocognitive and cardiovascular sequelae. Although the co-occurrence of SB and OSA has been described previously, the exact relationship remains unclear. The present study aimed to evaluate the incidence of SB in different phenotypes of OSA. (2) Methods: The participants of this study were adult patients referred to the Department and Clinic of Internal Medicine, Occupational Diseases, Hypertension and Clinical Oncology at the Wroclaw Medical University. They underwent a single-night video polysomnography in a sleep laboratory. The data related to common OSA phenotypes were analyzed in two separate groups of patients: body position related (*n* = 94) and rapid eye movement (REM) related (*n* = 85). (3) Results: The obtained results showed that the incidence of SB and severe SB was higher for body position-related OSA phenotype (*p* < 0.05 for all comparisons). No statistically significant differences were observed for REM-related OSA phenotype (*p* > 0.05 for all comparisons). (4) Conclusions: Body position-related OSA phenotype seems to be associated with higher SB and severe SB incidence, but the relationship is not independent. However, in the light of the unclear relationship between SB and sleep-disordered breathing, the topic needs further study.

## 1. Introduction

Sleep bruxism (SB) is defined “as a masticatory muscle activity during sleep that is characterized as rhythmic (phasic) or nonrhythmic (tonic) and is not a movement disorder or a sleep disorder in otherwise healthy individuals” [1]. It is estimated that SB affects approximately 13% of the global population. In fact, these data might be underestimated because many patients are unaware of their problem (sleep time occurrence), and access to objective instrumental methods of assessing this phenomenon is still limited in many countries [1,2,3,4,5]. Bruxism can be classified as a sleep-related motor behavior, a risk factor, or a protective factor [1,2]. However, in some individuals, for example, those with rapid eye movement (REM) sleep behavior disorder, epilepsy, gastroesophageal reflux disease or obstructive sleep apnea (OSA), SB can be potentially considered a sign of a general disorder [1,2,3,4,5]. Furthermore, as an increased masticatory muscle activity, SB can lead to numerous clinical consequences such as damage to the dental hard tissues and oral mucosa, fatigue and pain in masticatory muscles, prosthodontic treatment failures, and/or headaches [1,2,3,4,5].

The relationship between SB and sleep-related breathing disorders (SRBDs) has been widely discussed in medical literature in recent years. In this field, the most discussed SRBD is OSA. OSA is a sleep-related respiratory disorder that is characterized by total (apnea) or partial (hypopnea) obstruction of airflow, usually leading to arousals as a response to respiratory events [6]. It affects approximately 8% to 9% of the general adult population [7,8,9]. Some authors suggest that the prevalence of OSA in the general population can be even higher [10]. OSA is also a major public health problem because of its association with many comorbidities [8,9,10,11]. 

The relationship between SB and OSA has been described through some hypotheses. First, both OSA and SB seem to be associated with arousals [1,2,3,4,5,6,7,8,9,10,11]. “Arousal is a period of sleep instability characterized by an abrupt variation of brain activity frequency without re-consciousness, following a sleep disruption” [12]. Considering the fact that SB is a motor response of the central nervous system to arousal [1,4,6] and OSA events lead to arousals [6], previous studies have demonstrated the co-occurrence of SB and OSA [13,14,15]. Second, an SB episode during the OSA event can be considered as a protective factor. An SB episode is believed to play an important role in reopening the upper airways during apnea and for preventing hypoxia [1,2,6]. Some of the available studies also suggested common therapeutic pathways for both SB and OSA, which indicate that appropriate OSA treatment could eliminate SB [16]. Although all the above-mentioned studies seem to indicate a cause-and-effect relationship between SB and OSA, none of them have actually directly supported it. Currently, all these studies indicate the co-occurrence of these two phenomena. Systematic reviews performed on the topic of the relationship between SB and OSA concluded that there is inadequate scientific evidence to define a clear link between SB and OSA [17,18,19].

In our previous studies, we conducted investigations to establish the relationship between SB and sleep breathing events. In the first study, we reported that in adult patients who underwent audio–video polysomnography (avPSG), the relationship between OSA and SB depended on the degree of severity of OSA. OSA was correlated with SB in mild and moderate cases of OSA in the group of patients with increased risk of OSA [20]. In the second study, we reported that tonic muscle contractions during SB episodes can be the cause and effect for the formation of respiratory events. This indicated that the occurrence of tonic SB episodes may be the key to understanding the causal relationship between SB and SRBD [21]. We also reported the relationship between SB and snoring [22,23]. Moreover, we reported that genetic polymorphisms occurring within the genes of the serotonin pathways might contribute to the association between SB and OSA [24]. All these research results showed that SB and OSA are concomitant conditions, and both were found to be related to sleep stage and body position [20,21,22,23,24]. This indicated the need to conduct a study to determine whether the incidence of SB is related to the OSA phenotype using the “gold standard” method in both SB and OSA diagnostics—avPSG. Therefore, the present study aimed to evaluate the incidence of SB in different OSA phenotypes. The research hypothesis assumed that the incidence of SB may be related to a specific OSA phenotype.

## 2. Materials and Methods

Information on participant recruitment and inclusion criteria, avPSG details, parameters, and scoring has been partially previously described in our already published studies [20,21,22,23,24,25,26].

### 2.1. Participants

The participants of this study were 110 adult Caucasians—patients of the Department and Clinic of Internal Medicine, Occupational Diseases, Hypertension and Clinical Oncology at the Wroclaw Medical University referred due to a suspicion of OSA in 2019 and 2020. The study was approved by the Ethical Committee of the Wroclaw Medical University (ID: KB-794/2019) and conducted in accordance with the Declaration of Helsinki. All study participants provided written informed consent to be included in this study. Information regarding clinical trial registration is available at www.ClinicalTrials.gov(identifier: NCT04214561); (accessed on 1 June 2022). 

### 2.2. Inclusion and Exclusion Criteria

We assumed the following inclusion criteria: age above 18, clinical suspicion of OSA, and agreement to take part in this study.

We also assumed the following exclusion criteria: severe systemic (especially neurological and mental, including genetic) disorders and diseases; active inflammation; active malignancy; pregnancy and confinement; treatment with or addiction to any analgesic agents and/or medications and drugs affecting the function of the nervous system, muscles, and breathing; and lack of agreement to take part in this study.

### 2.3. Polysomnography

All the included patients underwent a single-night avPSG with the use of NoxA1 (NOX Medical, Reykjavík, Iceland) device in the Sleep Laboratory operating in the Department and Clinic of Internal Medicine, Occupational Diseases, Hypertension and Clinical Oncology at Wroclaw Medical University. All the recordings were conducted considering the sleeping habits and preferences of the participants, between 22.00 and 06.00. The electrodes were arranged in a standard manner in accordance with the recommendation of the American Academy of Sleep Medicine (AASM). The one exception was placing bipolar leads for electromyographic recording bilaterally in the area of the origin and insertion of the masseter muscle.

The standard elements of avPSG were examined: audio and video recording; electrocardiographic, electroencephalographic, electrooculographic and electromyographic (from the chin and masseter muscles areas) recordings; abdominal and thoracic breathing activity; and body position. A NONIN WristOx2 3150 pulse oximeter (Nonin Medical Inc., Plymouth, MN, USA) was used to record the saturation level, pulse, and plethysmographic data. To restore the full avPSG recording, the Noxturnal software (Nox Medical, Reykjavík, Iceland) was used. An experienced and qualified physician scored and analyzed the avPSG recordings in 30 s epochs in accordance with the 2013 AASM standard criteria for sleep scoring [27].

#### 2.3.1. Breathing Parameters

Abnormal respiratory events were scored from the pressure airflow signal evaluated in accordance with the standard criteria of the AASM Task Force [27]. As mentioned above, the arterial oxygen saturation (SpO2) was measured with finger pulse oximetry. Apneas were defined as “the absence of airflow for ≥10 s”. Hypopnea was defined as “a reduction in the amplitude of breathing by ≥30% for ≥10 s with a ≥3% decline in blood oxygen saturation or subsequent arousal”. OSA was classified as none/minimal with apnea/hypopnea index (AHI) < 5 per hour, mild with AHI ≥ 5, but <15 per hour, moderate with AHI ≥ 15, but <30 per hour and severe with AHI ≥ 30 [28].

#### 2.3.2. SB Parameters

SB was assessed using audio and video recordings and bilateral masseter electromyography (EMG). The following indices were assessed: bruxism episodes index (BEI), phasic bruxism (more than three cyclic phasic EMG increase lasting 0.25–2 s), tonic bruxism (episodes lasting > 2 s), and mixed bruxism. The new SB episodes were scored when the activity was at least twice the amplitude of the background electromyography and after at least 3 s of stable electromyography [6,27]. SB was classified on the basis of number of bruxism episodes per hour of sleep (BEI) as: irrelevant (BEI < 2), mild to moderate (BEI 2–4), or severe (BEI > 4) [6].

#### 2.3.3. Sleep Parameters

The following sleep parameters were assessed: total sleep time (TST), sleep latency (SL), rapid eye movement (REM), rapid eye movement latency (REML), wake after sleep onset (WASO), sleep efficiency (SE), non-REM 1 sleep stage (N1), non-REM 2 sleep stage (N2), non-REM 3 sleep stage (N3), and arousals using the 2013 AASM standard criteria for sleep scoring [27].

### 2.4. Obstructive Sleep Apnea Phenotypes

The following types of OSA phenotypes were used for the purpose of the study: REM related and body position related. REM-related OSA was defined as sleep breathing abnormality in which apneas and hypopneas are confined mainly to REM sleep periods (defined as a total AHI(/h) ≥ 5 and rem AHI(/h)/non-rem AHI(/h) ≥ 2 with at least 30 min of REM sleep) [29,30]. Body position-related OSA was defined as a sleep breathing abnormality in which apneas and hypopneas are confirmed mainly in the supine position (defined as a total AHI(/h) ≥ 5 and supine AHI(/h)/non-supine AHI(/h) ≥ 2) [31].

### 2.5. Qualification of the Participants into Particular Data Analyses

**Figure 1:**
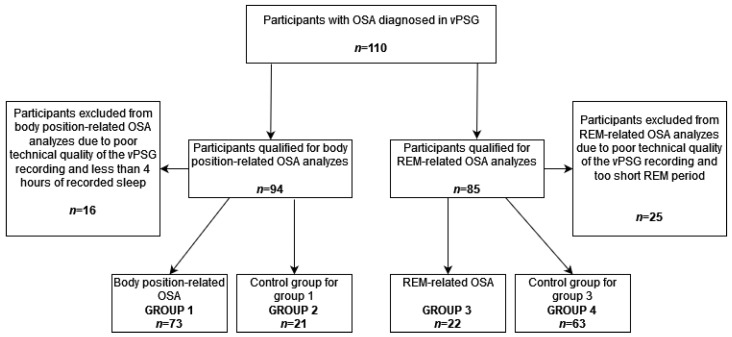
The scheme of study participants qualification to particular study groups. OSA—obstructive sleep apnea, REM—rapid eye movement, vPSG—video polysomnography.

At the beginning, 110 participants with OSA diagnosed in vPSG were included in this study. In accordance with the fact that specific OSA phenotypes can be diagnosed based on vPSG data only, polysomnographic data were then analyzed taking into account the criteria of body position-related and REM-related OSA. A total of 94 participants were included in the analysis regarding body position-related OSA. Overall, 16 study participants were excluded from this analysis due to the poor technical quality of the vPSG recording and less than 4 h of recorded sleep. In this group, 73 participants met the criteria for body position-related OSA (group 1) and 21 did not (group 2—control group for group 1). Additionally, among all study participants, 85 participants were included in the analysis regarding REM-related OSA. Moreover, 25 study participants were excluded from this analysis due to the poor technical quality of the vPSG recording and a too short period of REM sleep. In this group, 22 participants met the criteria of REM-related OSA (group 3) and 63 did not (group 4—control group for group 3). The scheme of qualifying the study participants to particular groups is presented in Figure 1.

### 2.6. Data Analysis

Results were considered significant at *p* < 0.05. During the statistical analysis, the use of parametric methods was initially preferred. Student’s *t*-test for parametric data and Mann–Whitney U test for nonparametric data were performed to test the significance in differences in the mean values between the groups. The data distribution shapes and deviations from the shape of the normal distribution were analyzed by the Shapiro–Wilk test. The qualitative variables were compared between the groups according by the Chi^2 test. Correlation analysis was performed using Spearman’s rank correlation test. Correlation and regression analyses were performed to determine the relationship between the studied variables. The group size was checked on the basis of the sample size calculator. The conditions for calculating the group size were as follows: population size 3 million, fraction size 0.5, maximum error 10%, and confidence level 95%. The required minimum size of the study group was 92. In the statistical analysis, both univariate and multivariate analyzes were performed. In the statistical analyzes, the conditions enabling the application of both types of analyzes were met. The group size was checked on the basis of the sample size calculator. The size of the group was sufficient for the types of analyzes performed. The selection of the statistical tests used took into account a specific group size. Statistica 13.1 (Statsoft, Cracow, Poland) program was used to analyze the obtained data.

## 3. Results

### 3.1. Body Position-Related OSA

The data of 94 study participants [60 men (63.83%) and 34 women (36.17%)] were qualified for comparative analyses in the field of body position-related OSA. The mean age of all participants was 52.19 ± 13.39 years. In total, 19 participants presented a normal BMI (20.21%), 33 were overweight (35.11%), and 40 were obese (42.55%). Moreover, 10 participants had diabetes (10.64%), 45 participants had hypertension (47.87%), and 7 participants had ischemic heart disease (7.45%).

OSA was classified as mild in 33 participants (35.11%), moderate in 27 participants (28.72%), and severe in 34 participants (36.17%). No SB was observed in 41 participants (43.62%), mild/moderate SB was observed in 22 participants (23.40%), and severe SB was observed in 31 participants (32.98%).

Among the participants, 73 (77.66%) met the criteria for body position-related OSA (group 1) and 21 (22.34%) did not (group 2). The supine AHI/non-supine AHI parameter was significantly higher in group 1 (*p* = 0.01) than in group 2. Group 1 also showed significantly lower BMI; AHI; obstructive apnea (OA); oxygen desaturation index (ODI); SpO2 < 90%; average desaturation drop, non-REM AHI, and non-supine AHI; and higher average SpO2 and mixed apnea (MA) (*p* < 0.05 for all the comparisons) (Table 1).

There were no differences in the incidence of mild, moderate, and severe OSA between group 1 and group 2 (*p* > 0.05 for all comparisons). The incidence of SB (BEI ≥ 2) and severe SB (BEI > 4) was significantly higher in body position-related OSA (*p* < 0.05 for both comparisons) (Table 2).

### 3.2. REM-Related OSA

The data of 85 study participants [57 men (67.06%) and 28 women (32.94%)] were qualified for comparative analyses for REM-related OSA. The mean age of all participants was 51.95 ± 13.39 years. Among them, 19 participants showed normal BMI (22.35%), 29 were overweight (34.12%), and 35 were obese (41.18%). Ten participants had diabetes (11.76%), 40 participants had hypertension (47.06%), and 7 participants had ischemic heart disease (8.24%).

OSA was classified as mild in 31 participants (26.47%), as moderate in 25 participants (28.41%), and as severe in 29 participants (34.12%). No SB was observed in 37 participants (43.53%), mild/moderate SB was observed in 20 participants (23.53%), and severe SB was observed in 28 participants (32.94%). 

Among the participants, 22 (25.88%) met the criteria of REM-related OSA (group 3) and 63 (74.12%) did not (group 4). The REM AHI/non-REM AHI parameter was significantly higher in group 3 (*p* = 0.00) than in group 4. Group 3 also showed significantly lower AHI, N1, ODI, Average Desaturation Drop, non-REM AHI, and supine AHI (*p* < 0.05 for all the comparisons) (Table 3).

A significantly lower incidence of severe OSA (AHI>30) was observed in group 3 (*p* < 0.05). There were no differences in SB incidence between groups 3 and 4 (*p* > 0.05 for all comparisons).

### 3.3. Regression Analysis

Additionally, we decided to conduct a regression analysis concerning body position-related OSA as a predictor of SB. As other predictors we analyzed: gender, age, BMI, diabetes, hypertension, and OSA severity. Multiple regression analysis showed that body position-related OSA was not an independent predictor of higher BEI values (*p* = 0.60). The independent predictors were male gender (*p* = 0.03) and diabetes (*p* = 0.04) (Table 4). What is more, logistic regression analysis showed no independent predictors of a higher probability of significant bruxism (BEI ≥ 2) (*p* > 0.05 for all comparisons). Only diabetes was an independent predictor for a higher probability of severe bruxism (BEI > 4) (*p* = 0.02).

## 4. Discussion

In the present study, we decided to focus on the incidence of SB in two OSA phenotypes: body position-related and REM-related OSA. The most important results obtained in this study showed that both SB (BEI ≥ 2) and severe SB (BEI > 4) were significantly more frequent in body position-related OSA. No significant differences in SB incidence were observed between REM-related and non-REM-related OSA patients. Moreover, the incidence of mild, moderate, and severe OSA differed when only REM-related OSA was considered. A significantly lower incidence of severe OSA (AHI > 30) was observed in patients with REM-related OSA. Unfortunately, regression analysis showed no independent relationship between OSA phenotypes and SB. As there are no other studies on the incidence of SB in body position-related and REM-related OSA, we discussed the potential causes of the reported associations in light of the existing literature focusing on similar topics.

OSA is a prevalent SRBD with potential long-term major neurocognitive and cardiovascular sequelae [32]. Its pathophysiology varies between individuals. It is estimated that several components influence the pathogenesis of OSA, including the anatomy of the upper airway, effectiveness of muscles, and stability of the respiratory control system [32]. The main risk factors for OSA are gender (men are at a higher risk), age (the risk increases with age), race (potentially due to cranial anatomy), and obesity (increased BMI increases the risk of OSA) [8]. However, it should be noted that OSA seems to be underdiagnosed in females [33]. As OSA is a very heterogenous condition, the heterogeneity of OSA pathophysiology is more recognizable in patients with mild and moderate OSA than in patients with severe OSA [34]. As mentioned in the Introduction section, OSA is highly associated with arousals [12,13]. Arousals are also considered a risk factor for SB [1,4,6] and seem to bind both of these phenomena, but the exact cause-and-effect relationship remains unclear [15,16,17]. Our team previously published studies that led us to hypothesize that the occurrence of SB may vary depending on the OSA phenotype [20,21,22,23,24,25,26]. Therefore, the present study aimed to evaluate the incidence of SB in different OSA phenotypes.

Martynowicz et al. focused on the relationship between OSA and SB and observed that BEI was increased in the group with mild and moderate OSA compared to that in the group with severe OSA [20]. The former group was previously described as more heterogenous when the heterogeneity of OSA pathophysiology was considered [34]. Martynowicz et al. reported a positive correlation between AHI and BEI in the group of patients with non-severe OSA. The independent predictors for the increased BEI in nonsevere OSA patients were AHI, male gender, and diabetes [20]. As patients with mild and moderate OSA seem to be a more heterogenous group, it is estimated that the pathophysiology of OSA in this group is more likely to be body position or REM related [32]. Michalek-Zrabkowska et al. reported that the supine sleep position increases SB intensity [22]. SB episodes were also reported to not only co-occur with OSA episodes (considering arousals as a linking factor) but also to be a protective factor, leading to the restoration of proper airflow in the upper airway [1,4,6,7,8,9,12,35]. It has also been reported that body position during sleep influences upper airway patency [35] and supine sleep position increases OSA due to anatomical features [36,37]. On the basis of this information, it could be hypothesized that the higher incidence of both SB and severe SB associated with body position-related OSA (supine position) reported in the present study can occur due to the anatomical reduction of airflow in the supine position. In that case, SB episodes could occur as a protective factor.

The present study showed no differences in SB incidence for REM-related OSA. REM-related OSA was reported to be associated with age and gender and were found to be more common in women and younger patients [30]. However, these features are not considered as general risk factors for OSA [8]. As previously described, an increased risk of OSA is associated with the male gender and increased age [8]. Moreover, in the present study, REM-related OSA was associated with generally lower AHI and supine AHI scores, and lower incidence of severe OSA. When considering an SB episode as the protective factor that restores proper airflow through the upper airway during an OSA episode [1,4,6] and the fact that it was reported to be associated with supine sleep position [22] and increased AHI [20], the results of the present study seem to confirm the previously described dependencies. It can also be explained by the fact that SB was generally reported to occur more frequently (80%) in N1 and N2 sleep stages [6]. Summarizing, REM-related OSA is less expected to co-occur with bruxism due to related predictors. Considering SB as a protective factor in hypoxia caused by OSA episodes, the hypothesis that SB episodes should appear in the same mechanism for REM-related and body position-related OSA appears [1,4,6]. However, the relationship with SB in the presented study was demonstrated only for the body position-related OSA. This may be because of the fact that the REM sleep stage is only a relatively small portion of the general sleep period. It means that individuals with REM-related OSA will generally have lower AHI scores than individuals with body position-related OSA [38]. What is more, a lower AHI score will potentially lower the need for SB to appear as a protective factor.

To the best of our knowledge, this is the first study to evaluate the incidence of SB in different OSA phenotypes. It was also conducted on a large population and used the most available and objective method of SB and OSA diagnosis (avPSG). The results of the present study are novel and constitute a link between our previously published studies. Despite showing very promising results, the present study has the following limitations: (1) only one-night avPSG examination was conducted because of the restrictions of the Polish healthcare system, and (2) the present study was exploratory in nature, which means that the results should be supported with further research. The possible clinical implication regarding the results obtained in this study could be a need for more thorough dental care of patients with body position-related OSA. Furthermore, it is recommended to include SB diagnostics as a permanent element of OSA patients’ polysomnographic assessment.

## 5. Conclusions

The body position-related OSA phenotype seems to be associated with higher SB and severe SB incidence, but the relationship between the OSA phenotype and SB is not independent. Moreover, in light of the unclear relationship between SB and SRBD, the topic needs to be further studied, focusing on establishing the cause-and-effect relationship between SB and OSA.

## Figures and Tables

**Table 1:** Quantitative comparison of the parameters in groups 1 (meeting the criteria for body position-related OSA) and 2 (not meeting the criteria for body position-related OSA—control group).

	Group 1 (n = 73)	Group 2 (n = 21)	*p*
Gender ^a^ ^			0.46
Male	65.7 (48)	57.1 (12)	
Female	34.3 (25)	42.8 (9)	
BMI (kg/m^2^) ^b^ *	28.43 (5.40)	31.60 (4.43)	0.02
Body mass ^a^ ^			0.02
Normal	25.0 (18)	5.0 (1)	
Overweight	38.9 (28)	25.0 (5)	
Obesity	36.1 (26)	70.0 (14)	
Age (years) ^b #^	51.25 (14.08)	55.48 (10.23)	0.20
AHI (/h) ^b^ *	23.62 (15.60)	37.03 (27.64)	0.01
SL (min) ^b^ *	20.29 (19.49)	22.12 (21.07)	0.71
WASO (min) ^b^ *	55.89 (40.05)	63.97 (38.73)	0.41
SE (%) ^b^ *	81.26 (10.17)	79.98 (11.16)	0.62
N1 (%TST) ^b^ *	6.17 (4.62)	6.57 (6.42)	0.75
N2 (%TST) ^b #^	48.02 (9.45)	48.51 (12.66)	0.85
N3 (%TST) ^b #^	23.61 (9.35)	23.70 (10.42)	0.97
REM (%TST) ^b #^	22.20 (7.65)	21.23 (9.62)	0.63
Arousals (/h) ^b^ *	6.59 (5.48)	7.96 (13.28)	0.48
OA (/h) ^b^ *	6.85 (10.10)	15.35 (23.67)	0.02
MA (/h) ^b^ *	0.14 (0.45)	1.79 (5.69)	0.01
CA (/h) ^b^ *	0.74 (2.00)	1.53 (3.34)	0.18
ODI/h ^b^ *	23.11 (16.17)	36.22 (25.86)	0.01
Average SpO_2_ (%) ^b^ *	92.77 (1.84)	91.48 (3.12)	0.02
Minimal SpO_2_ (%) ^b^ *	81.44 (6.11)	78.10 (10.37)	0.07
SpO_2_ < 90% (%) ^b^ *	11.22 (17.16)	21.02 (26.81)	0.05
Average Desaturation Drop (%) ^b^ *	4.70 (1.56)	6.06 (3.97)	0.02
BEI (/h) ^b^ *	4.26 (4.44)	3.12 (4.69)	0.31
Phasic Bruxism (/h) ^b^ *	2.18 (3.15)	1.98 (3.90)	0.81
Tonic Bruxism (/h) ^b^ *	1.33 (1.48)	0.75 (0.78)	0.09
Mixed Bruxism (/h) ^b^ *	0.78 (0.87)	0.45 (0.59)	0.11
TST (min) ^b^ #	414.04 (58.07)	407.26 (65.14)	0.65
REM Latency (min) ^b^ *	96.60 (59.09)	92.55 (56.57)	0.78
REM AHI (/h) ^b^ *	26.04 (19.88)	36.06 (27.19)	0.07
Non-REM AHI (/h) ^b^ *	22.56 (16.15)	37.68 (29.88)	0.00
Supine AHI (/h) ^b^ *	43.72 (29.65)	39.77 (32.80)	0.60
Non-supine AHI (/h) ^b^ *	9.74 (10.44)	35.15 (27.25)	0.00

^a^—qualitative variable expressed as a percentage (number of cases); ^b^—quantitative variable expressed as a mean (standard deviation); ^—comparison of means tested with Chi2 test; ^#^—comparison of means tested using the *t*-test due to the normal distribution of the variable; *—comparison of means tested using the Mann–Whitney U test due to the lack of a normal distribution of the variable BMI—body mass index, AHI—Apnea/Hypopnea Index, /h—per hour, SL—sleep latency, WASO—Wake After Sleep Onset, SE—Sleep Efficiency, OA—Obstructive Apnea, CA—Central Apnea, MA—Mixed Apnea, ODI—Oxygen Desaturation Index, BEI—Bruxism Episode Index, TST—Total Sleep Time, SD—standard deviation, REM—rapid eye movement sleep stage, N1—non-REM 1 sleep stage, N2—non-REM 2 sleep stage, N3—non-REM 3 sleep stage.

**Table 2:** Comparison of the parameters in groups 1 (meeting the criteria of body position-related OSA) and 2 (not meeting the criteria of body position-related OSA—control group) regarding occurrence of SB (BEI ≥ 2) and severe SB (BEI > 4).

Parameter	Group 1		Group 2		*p*
	*n*	%	*n*	%	
BEI < 2	29	40	12	57	*p* < 0.05
BEI ≥ 2	44	60	9	43	
BEI ≤ 4	46	63	17	81	
BEI > 4	27	37	4	19	

BEI—Bruxism Episode Index, N—number of participants.

**Table 3:** Quantitative comparison of the parameters in groups 3 (meeting the criteria of REM-related OSA) and 4 (not meeting the criteria of REM-related OSA—control group).

	Group 3 (n = 22)	Group 4 (n = 63)	*p*
Gender ^a^ ^			0.05
Male	50.0 (11)	73.0 (46)	
Female	50.0 (11)	27.0 (17)	
BMI (kg/m^2^) ^b^ *	28.59 (6.83)	29.23 (5.13)	0.65
Body mass ^a^ ^			0.52
Normal	27.3 (6)	21.3 (13)	
Overweight	40.9 (9)	32.8 (20)	
Obesity	31.8 (7)	45.9 (28)	
Age (years) ^b #^	49.68 (15.35)	52.75 (12.67)	0.36
AHI (/h) ^b^ *	17.76 (10.78)	29.68 (21.70)	0.02
SL (min) ^b^ *	18.17 (17.12)	21.94 (21.32)	0.46
WASO (min) ^b^ *	52.70 (38.39)	60.14 (41.12)	0.46
SE (%) ^b^ *	83.50 (8.49)	80.14 (11.01)	0.20
N1 (%TST) ^b^ *	3.89 (3.63)	7.24 (5.07)	0.01
N2 (%TST) ^b #^	47.44 (10.63)	48.61 (9.91)	0.64
N3 (%TST) ^b #^	26.36 (12.69)	22.90 (8.56)	0.16
REM (%TST) ^b #^	22.33 (6.86)	21.25 (7.35)	0.55
Arousals (/h) ^b^ *	4.31 (3.16)	8.09 (9.12)	0.06
OA (/h) ^b^ *	4.93 (6.16)	10.62 (17.03)	0.13
MA (/h) ^b^ *	0.02 (0.07)	0.73 (3.35)	0.32
CA (/h) ^b^ *	0.22 (0.44)	1.16 (2.78)	0.12
ODI/h ^b^ *	17.17 (10.91)	29.06 (21.28)	0.01
Average SpO_2_ (%) ^b^ *	92.66 (1.65)	92.52 (2.34)	0.79
Minimal SpO_2_ (%) ^b^ *	82.91 (6.75)	80.13 (7.64)	0.13
SpO_2_ < 90% (%) ^b^ *	7.84 (14.32)	13.71 (19.84)	0.21
Average Desaturation Drop (%) ^b^ *	4.13 (1.10)	5.35 (2.73)	0.05
BEI (/h) ^b^ *	4.40 (4.17)	4.03 (4.82)	0.75
Phasic Bruxism (/h) ^b^ *	2.61 (3.17)	2.10 (3.55)	0.55
Tonic Bruxism (/h) ^b^ *	1.17 (1.45)	1.20 (1.38)	0.93
Mixed Bruxism (/h) ^b^ *	0.68 (0.82)	0.76 (0.88)	0.71
TST (min) ^b^ #	422.92 (50.75)	408.24 (62.96)	0.33
REM Latency (min) ^b^ *	96.25 (51.51)	107.12 (55.57)	0.42
REM AHI (/h) ^b^ *	34.21 (19.05)	26.52 (23.50)	0.17
Non-REM AHI (/h) ^b^ *	12.44 (8.74)	30.46 (22.58)	0.00
Supine AHI (/h) ^b^ *	30.85 (24.82)	46.04 (31.26)	0.04
Non-supine AHI (/h) ^b^ *	9.02 (7.73)	17.82 (21.49)	0.06

^a^—qualitative variable expressed as a percentage (number of cases); ^b^—quantitative variable expressed as a mean (standard deviation); ^—comparison of means tested with Chi2 test; ^#^—comparison of means tested using the *t*-test due to the normal distribution of the variable; *—comparison of means tested using the Mann-Whitney U test due to the lack of a normal distribution of the variableBMI—body mass index, AHI—Apnea/Hypopnea Index, /h—per hour, SL—sleep latency, WASO—Wake After Sleep Onset, SE—Sleep Efficiency, OA—Obstructive Apnea, CA—Central Apnea, MA—Mixed Apnea, ODI—Oxygen Desaturation Index, BEI—Bruxism Episode Index, TST—Total Sleep Time, SD—standard deviation, REM—rapid eye movement sleep stage, N1—non-REM 1 sleep stage, N2—non-REM 2 sleep stage, N3—non-REM 3 sleep stage.

**Table 4:** Multiple regression analysis for independent predictors of higher BEI values.

Predictor	β	Standard Error with β	b	Standard Error with b	t (83)	*p*
Body position-related OSA	0.059	0.112	0.642	1.231	0.521	0.604
Gender—male (0)/female (1)	−0.246	0.113	−2.328	1.072	−2.171	0.033
Body mass index	−0.068	0.119	−0.058	0.101	−0.576	0.566
Age	−0.085	0.117	−0.029	0.040	−0.727	0.469
Diabetes	0.242	0.118	3.508	1.714	2.047	0.044
Hypertension	−0.129	0.117	−1.167	1.055	−1.106	0.272
Ischemic heart disease	−0.101	0.119	−1.723	2.034	−0.847	0.399
Apnea/Hypopnea Index	−0.148	0.128	−0.034	0.030	−1.162	0.249

β—beta statistic of the estimator; b—regression coefficient.

## Data Availability

The data that support the findings of this study are available on request from the corresponding author and are not publicly available due to privacy or ethical restrictions.
